# Indoor Light Harvesting Perovskite Solar Cells on Conducting Oxide-Free Ultrathin Deformable Substrates

**DOI:** 10.1021/acsaem.3c02581

**Published:** 2024-07-22

**Authors:** Arivazhagan Valluvar Oli, Aruna Ivaturi

**Affiliations:** Smart Materials Research and Device Technology Group, Department of Pure and Applied Chemistry, University of Strathclyde, Glasgow G1 1XL, U.K.

**Keywords:** perovskite solar cells, indoor energy harvesting, stretchable substrate, compressive strain, wearable devices

## Abstract

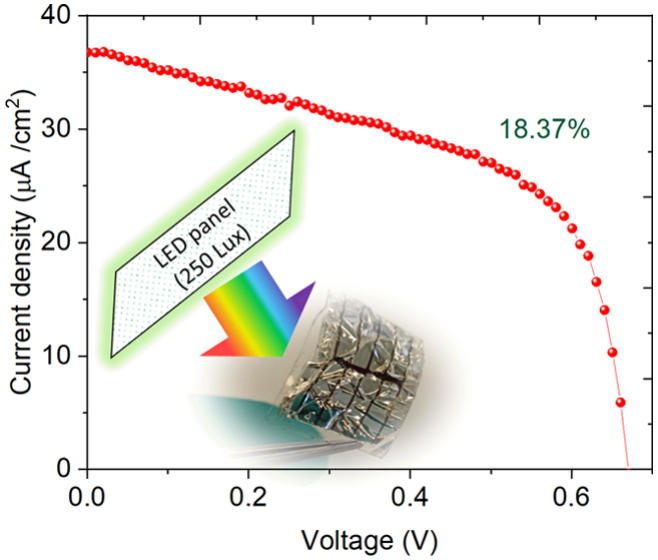

Perovskite solar cells (PSCs) are receiving renewed interest since they have reached high power conversion efficiency (PCE) and show potential for application not only on rigid and flexible substrates but also on mechanically deformable substrates for integration on nonplanar curvilinear surfaces. Here we demonstrate PSCs fabricated on transparent conducting oxide-free ultrathin polyethylene terephthalate substrates capable of efficiently harvesting indoor light even under compressive strain. Interface engineering with poly(bis(4-phenyl)(2,4,6-trimethylphenyl)amine) improved the shunt resistance and band alignment at the perovskite-hole transport layer interface, which resulted in enhanced charge extraction, leading to 114% improvement in PCE from 5.57 to 11.91% under 500 lx indoor white LED (4000 K) illumination. The champion device exhibited a PCE of 18.37% under 250 lx cool white LED (4000 K) light. The maximum power output (*P*_max_) of the devices varied from 13.78 to 25.38 μW/cm^2^ by changing the indoor light illumination from 250 to 1000 lx, respectively. Moreover, the devices showed impressive performance even after mechanical deformation and retained 83 and 76% for 1 sun and indoor light, respectively, under 30% compressive strain. Our approach paves the way for fabrication of efficient indoor light harvesting PSCs on mechanically deformable substrates for integration on nonplanar surfaces prone to compressive strain.

## Introduction

Perovskite solar cells (PSCs) have attracted great attention due to their promising commercial prospects as the certified power conversion efficiency (PCE) exceeding 26.1% has already been achieved in single junction solar cells fabricated on transparent conducting oxide (TCO)-coated rigid glass substrates.^1^ The tremendous breakthroughs in PSCs have been achieved due to the outstanding optoelectronic properties of the perovskite absorber, such as panchromatic absorption, long charge diffusion length, tunable band gap, and low exciton binding energy.^2−5^ Besides the solar cell fabrication on rigid substrates, PSCs on flexible substrates are receiving more attention for their lightweight, ease of integration on curved surfaces, low-temperature solution processability, and compatibility with well-established roll-to-roll technology.^6−10^ While the mechanical compliance of PSCs fabricated on flexible substrates such as poly(ethylene terephthalate) (PET) is remarkable, these substrates, however, cannot be used under mechanical deformation such as stretching and compression. In contrast to just flexible PET substrates, mechanically deformable substrates such as polydimethylsiloxane (PDMS) offer additional advantages (such as compressibility and/or stretchability) over just bending deformation.^11−14^ Solar cells fabricated on such substrates can be potential power sources for a number of applications such as wearable electronics, electronic skins, stretchable displays, etc.^15−20^ However, it is challenging to deposit rigid TCO layers on such deformable substrates without compromising the mechanical compliance, and thus alternate transparent conducting layers are required. Poly(3,4-ethylenedioxythiophene):polystyrenesulfonate (PEDOT:PSS) is one of the widely studied alternative TCO-free conducting layers explored for several optoelectronic applications, including solar cells, due to its high transparency (>90%), conductivity (>4000 S/cm), and high work function (>5.0 eV).^21−23^ Further improvements in conductivity and work function have been achieved through doping strategies for high-performance PSCs. For example, methanesulfonic acid treatment on PEDOT:PSS led to PSC with PCE of 8.6% on a flexible PET substrate.^24^ Ethylene glycol and phosphoric acid treatments showed improved conductivity of PEDOT:PSS and showed 10.51% PCE for the PSC fabricated on flexible substrates.^25^ Slot-die-coated silver nanowire/PEDOT:PSS electrode on a flexible PET substrate delivered 11% PCE in PSC and showed negligible change in PCE after 1000 compressive bends to a 5 mm radius.^6^ Stretchable and lightweight flexible PSCs were realized using PEDOT:PSS doped with ZnTFSI or DMSO with Zonyl FS-30 fluorosurfactant transparent electrodes.^26,27^ Besides chemical doping and surface treatments, a simple oxygen plasma treatment on PEDOT:PSS films has also been reported to reduce the sheet resistance significantly and resulting in PCE of 10.5% in PSCs.^28^ Thus, PEDOT:PSS is an excellent choice to use as a transparent electrode on mechanically resilient substrates. Number of highly efficient organic solar cells have been reported on PDMS (stretchable) and PET (compressible) substrates;^18,29−32^ however, there are only a few reports on PSCs. For example, PSCs on stretchable PDMS substrates with the PCE of 19.15% have been reported in which polyurethane was used as an additive to enhance the crystallinity and passivate the grain boundaries in the perovskite thin film.^27^ Flexible and high-power-per-weight PSCs have been reported with chromium oxide-metal contacts for improved air stability with recoverable performance under compressive strain up to 44%.^26^ However, indoor energy harvesting PSCs on mechanically deformable substrates are not reported until date. For indoor energy harvesting PSCs, unlike 1 sun illumination, the charge extraction at the interfaces with high shunt resistance is paramount as the number of electron–hole pair generation from the indoor light is relatively less.^6,33,34^ Under 1 sun illumination, the carrier concentration is high, and the shunt path can be overcome rather quickly at the interface. Under indoor light, the carrier concentration is less; therefore, even a minor defect at the interface will lead to poor extraction, which results in poor device performance. In general, interface modifications with band alignment showed improved shunt resistance and charge carrier extraction,^35−37^ which can be decidedly applicable for indoor energy harvesting solar cells.

Here we demonstrate PSCs fabricated on TCO-free PET substrates that have ultraflexibility and mechanical deformability. The PSCs were fabricated in the p-i-n configuration with the device architecture of PDMS/PET/PH1000/AI4083/MAPbI_3_/PCBM/BCP/Ag. We employed PEDOT:PSS (PH1000) as the transparent conducting electrode as reported elsewhere.^6,27^ The interface between the hole transport layer (PEDOT:PSS, AI4083) and the perovskite (MAPbI_3_) was further improved with an optimal poly(bis(4-phenyl)(2,4,6-trimethylphenyl)amine) (PTAA) interlayer. The AI4083/PTAA double hole transport layer combination facilitates enhanced hole extraction as a result of better band alignment, which in turn improves the shunt resistance for indoor light energy harvesting. The current–voltage characteristics of the fabricated devices were measured under 1 sun and indoor LED at different illumination levels. The champion device with an optimized PTAA interlayer delivered a PCE and maximum power of 11.91% and 17.87 μW/cm^2^, respectively, under 500 lx white LED panel (4000 K). The devices showed a stable current density biased at maximum power point (MPP). Moreover, the performance of the solar cells under mechanical deformation of the substrates was investigated. The champion devices retained 83 and 76% for 1 sun and indoor light, respectively, under 30% compressive strain. This work paves the way for fabrication of indoor energy harvesting PCSs on mechanically deformable substrates for off-grid power source applications on nonplanar curvilinear surfaces.

## Experimental Section

The SYLGARD 184 Silicone Elastomer was mixed with cross-linker at a 10:1 weight ratio (in a vial), degassed for 30 min, and spin coated on a 7 × 7 cm FTO-coated glass substrate which was then annealed overnight at 80 °C in a convection oven to obtain about 100–120 μm thick layer. The PDMS was then peeled off from the FTO substrates and cut into the desired size and attached to a glass substrate (20 × 15 mm^2^). PET (1.4 μm, Dupont Teijin Films) was then carefully attached to the glass/PDMS substrate without any wrinkle formation and degassed in a vacuum chamber to remove any bubble formation underneath, followed by oxygen plasma treatment for 10 min. PET films were optically clear and durable with high thermal resistance. The conductive PEDOT:PSS precursor was prepared by mixing PH1000 with 5% v/v DMSO and 0.7% v/v capstone. The PH1000 precursor was then spin coated on PET substrates at 800 rpm for 50 s, annealed at 120 °C for 20 min, and patterned to leave an area of ∼12 × 15 mm^2^ of coated PEDOT:PSS. After the films were cooled down to room temperature, the PH1000 was flash plasma treated for 30 s (air plasma), then the hole transport PEDOT:PSS (AI4083) layer was deposited at 3000 rpm for 30 s and annealed at 140 °C for 15 min. The AI4083 surface was then flash plasma treated for 30 s. For the PTAA interlayer, 0.05–0.2 mg/mL PTAA dissolved in toluene was spin coated at 3000 rpm for 30 s and annealed at 100 °C for 10 min. The PTAA-coated substrates were then flash plasma treated for 20 s and transferred to a glovebox for perovskite deposition. The air plasma flash in each step is required for better wettability and coverage and to avoid intense plasma that could otherwise etch the organic film rather quickly. For MAPbI_3_, 642 mg of PbI_2_ and 222 mg of MAI were dissolved in 900 μL DMF:100 μL DMSO and stirred to get the clear solution. 50 μL of precursor solution was spin coated on the PTAA-coated substrates using three-step program: 1000 rpm for 5 s, 2000 rpm for 10 s, and 5000 rpm for 20 s (1 s to reach the required rpm in all the stages). At the beginning of the 5000 rpm step, 200 μL of chlorobenzene was dropped on the spinning substrate. The adduct film was annealed at 70 °C for 1 min and 100 °C for 10 min. For the electron transport layer, 40 μL of PCBM (20 mg of PCBM dissolved in 1 mL of chlorobenzene) was spin coated inside the glovebox at 3000 rpm for 30 s. Then bathocuproine (BCP, 0.5 mg/mL in 2-propanol) was spin coated at 4000 rpm for 30 s. The devices were completed by evaporating silver electrodes (110 nm) at the base pressure of 5 × 10^–7^ mbar through a shadow mask. The details of the material and device characterization techniques used are provided in the Supporting Information.

## Results and Discussion

**Figure 1:**
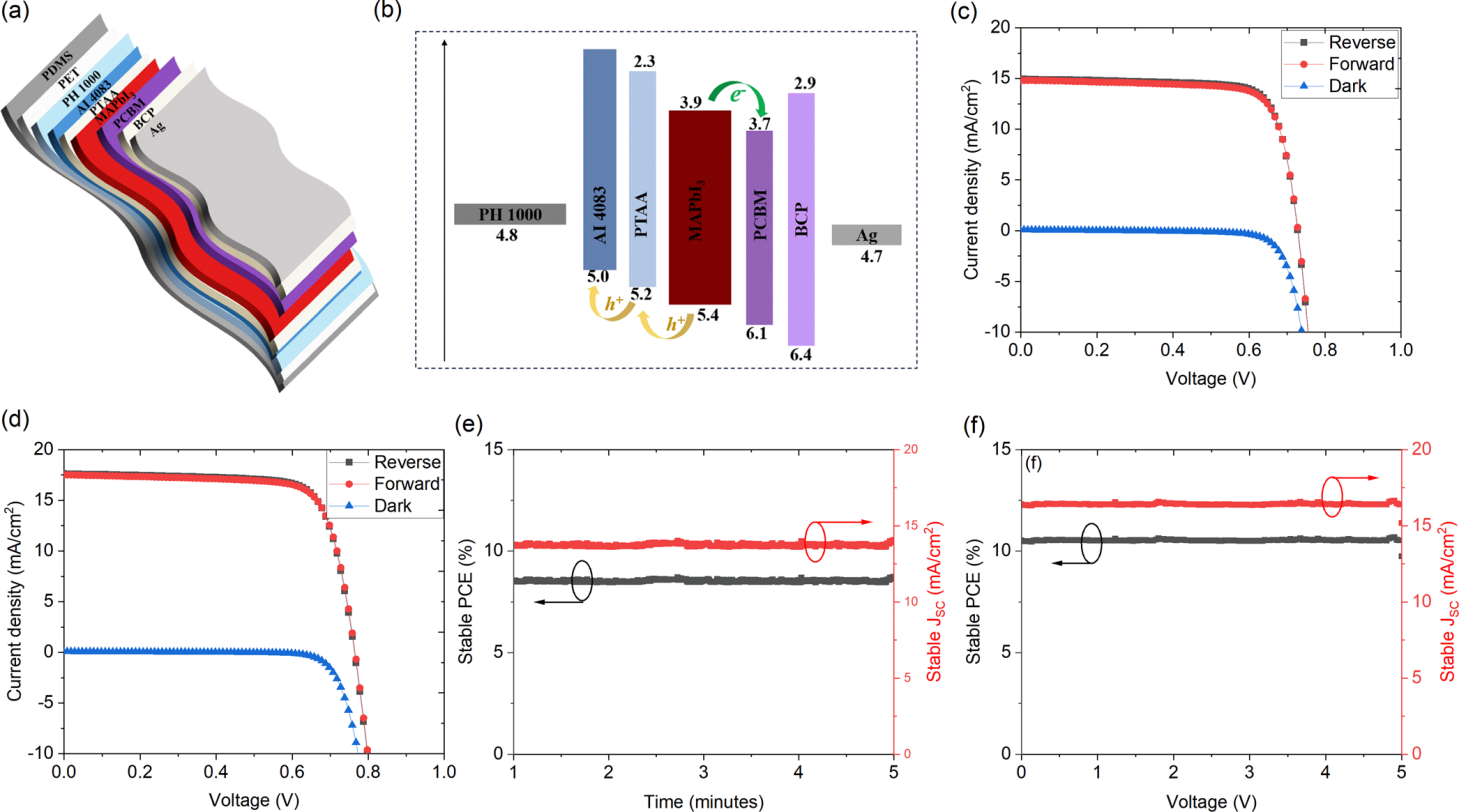
(a) Schematic of the solar cell on PET substrate, (b) band diagram, *J–V* curves of (c) control and (d) PTAA interlayer-based devices; stabilized power output of the (e) control and (f) PTAA interlayer devices biased at maximum power point. All of the *J–V* and MPP measurements are performed under 1 sun illumination.

The PSCs with the configuration of PET/PH1000/AI4083/MAPbI_3_/PCBM/BCP/Ag were fabricated with an optimized PTAA interlayer between the AI4083 and MAPbI_3_. The PTAA interlayer was optimized by varying the concentration from 0.05 to 0.2 mg/mL, and the resulting reverse scan *J–V* curves under AM1.5G 1 sun illumination are shown in Figure S1, and the corresponding photovoltaic parameters are summarized in Table S1. The optimal 0.1 mg/mL PTAA was used for detailed studies reported in this work. Figure 1a shows the schematic of the solar cells, where the PTAA interfacial layer was introduced between the AI4083 and MAPbI_3_. The corresponding band diagram of the solar cell is shown in Figure 1b. The dark, forward, and reverse scan *J–V* curves of the control and PTAA interlayer-based solar cells are shown in Figure 1c,d, respectively, and their photovoltaic parameters are summarized in Table 1. The PTAA interlayer improved the PCE from 8.47 to 10.25% with enhancement in both *J*_SC_ and *V*_OC_. In order to understand the operational stability of the PSCs on the PET substrate, the devices were biased at MPP over a period of 5 min, and the resulting stabilized *J*_SC_ and power output of control (MPP = 0.62 V) and PTAA interlayer (MPP = 0.64 V)-based devices are shown in Figure 1e,f. Both the devices showed negligible hysteresis and stable performance under 1 sun illumination. The box chart in Figure S2 shows the statistical distribution of the photovoltaic parameters of the 20 devices, where the narrow distribution indicates the reproducible performance. The EQE of the best performing device is shown in Figure S3.

**Table 1:** Photovoltaic Parameters of the Control and PTAA Interlayer-Based Devices Measured under 1 sun Illumination

devices	scan	PCE (%)	FF (%)	*J*_SC_(mA/cm^2^)	*V*_OC_ (V)
**control**	R	8.47	77.75	14.95	0.73
	F	8.34	77.15	14.83	0.73
**PTAA**	R	10.25	76.13	17.62	0.76
	F	10.18	75.94	17.51	0.77

**Figure 2:**
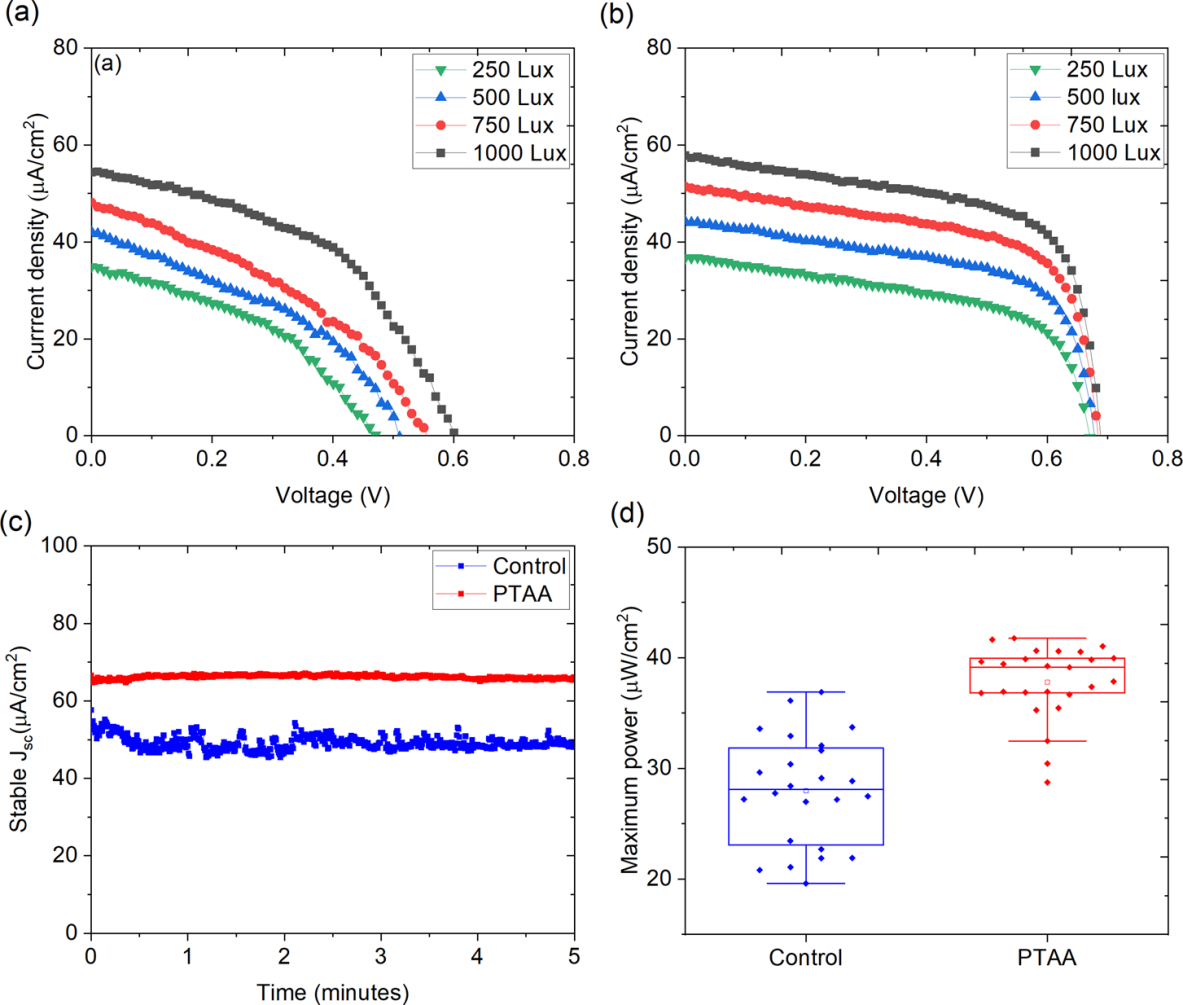
Reverse scan *J–V* curves of (a) control and (b) PTAA interlayer-based devices. (c) Stabilized current density of devices biased at MPP and (d) maximum power output of the control and PTAA interlayer-based devices illuminated under 1000 lx.

The *J–V* characteristics of the devices were then measured under an indoor LED panel (Rexel white light 36W, 34000 lm, 4000 K cool white) at different illumination levels. The ILT 350 spectrometer was used to measure the incident power on the device surface, and measurement details are given in the Supporting Information. Figure 2a,b shows the reverse scan *J–V* curves of the control and PTAA interlayer-based devices, respectively, under different illuminations. The illumination intensity was varied from 250 to 1000 lx. The champion device exhibited a PCE of 18.37% under 250 lx cool white LED (4000 K) light. The devices without a PTAA interlayer showed inferior *J–V* characteristics as a result of slow extraction of holes, as can be easily observed from the *J–V* curves. Upon the addition of the PTAA interlayer, the *J–V* characteristic improved with improved fill factor indicating accelerated charge extraction at the interface.^38^ The box plot of photovoltaic parameters of the devices showing the statistical distribution under 500 lx is given in Figure S4. Table 2 gives the photovoltaic parameters of the control and PTAA interlayer-based devices measured under 500 lx, and Table S2 gives the parameters obtained under various illuminations. Unlike 1 sun illumination, charge carrier generation under indoor LED is limited due to the intensity of the light. It is worthy to note that the interface between the perovskite and selective contact plays a vital role in extracting charge carriers and is even more critical when the excited charge carrier density is low.^39,40^ Here we note that the devices with the PTAA interlayer strengthened the band alignment between the perovskite and AI4083 (see Figure 1b) which obviously led to enhanced hole extraction from the perovskite layer, resulting in improved device performance. In general, the shunt resistance (*R*_sh_) at the interface needs to be high in order to extract the maximum charge carriers under dim light conditions.^41,42^ As summarized in Tables 2 and S2, the *R*_sh_ for the PTAA interlayer-based device is much higher than that for the control device, which is beneficial for the enhanced hole extraction. As a result, the device with the PTAA interlayer generated the maximum power output of 25.38 μW/cm^2^ under 1000 lx, which is 63.11% increase from the control device (15.56 μW/cm^2^). We note that PTAA also increases the series resistance; however, it does not affect the device performance significantly as the dominating carrier recombination is through the shunt path, which becomes high after PTAA. Figure 2c shows the stabilized *J*_SC_ of the devices under 1000 lx biased at MPP. It can be noted that the *J*_SC_ obtained from the MPP is 47.09 and 66.17 μA/cm^2^ for control and PTAA-based devices, respectively, which are higher than the *J*_SC_ obtained from the *J–V* scan (47.26 and 63.75 μA/cm^2^ for control and PTAA devices, respectively). We attribute this increase in *J*_SC_ to the light soaking effect. This is because charge carrier generation under indoor light with a single *J–V* scan is less, while under MPP, the density of the photogenerated carriers piles up at the conduction band minimum to deliver high *J*_SC_. Figure 2d shows the box chart of the maximum power output from over 13 devices under 1000 lx.

**Table 2:** Photovoltaic Parameters of the Control and PTAA Interlayer-Based Champion Devices Measured under 500 lx LED

device	scan	PCE (%)	*J*_SC_ (μA/cm^2^)	FF (%)	*V*_OC_ (V)	*P*_max_ (μW/cm^2^)	*R*_sh_(kΩ cm^2^)	*R*_s_(Ω cm^2^)
control	R	5.57	39.67	42.12	0.50	8.37	21.16	39.67
	F	5.32	33.61	42.11	0.56	7.99	21.24	33.61
**PTAA**	R	11.91	59.44	44.31	0.67	17.87	50.69	59.44
	F	11.55	57.89	44.12	0.67	17.33	56.524	57.89

**Figure 3:**
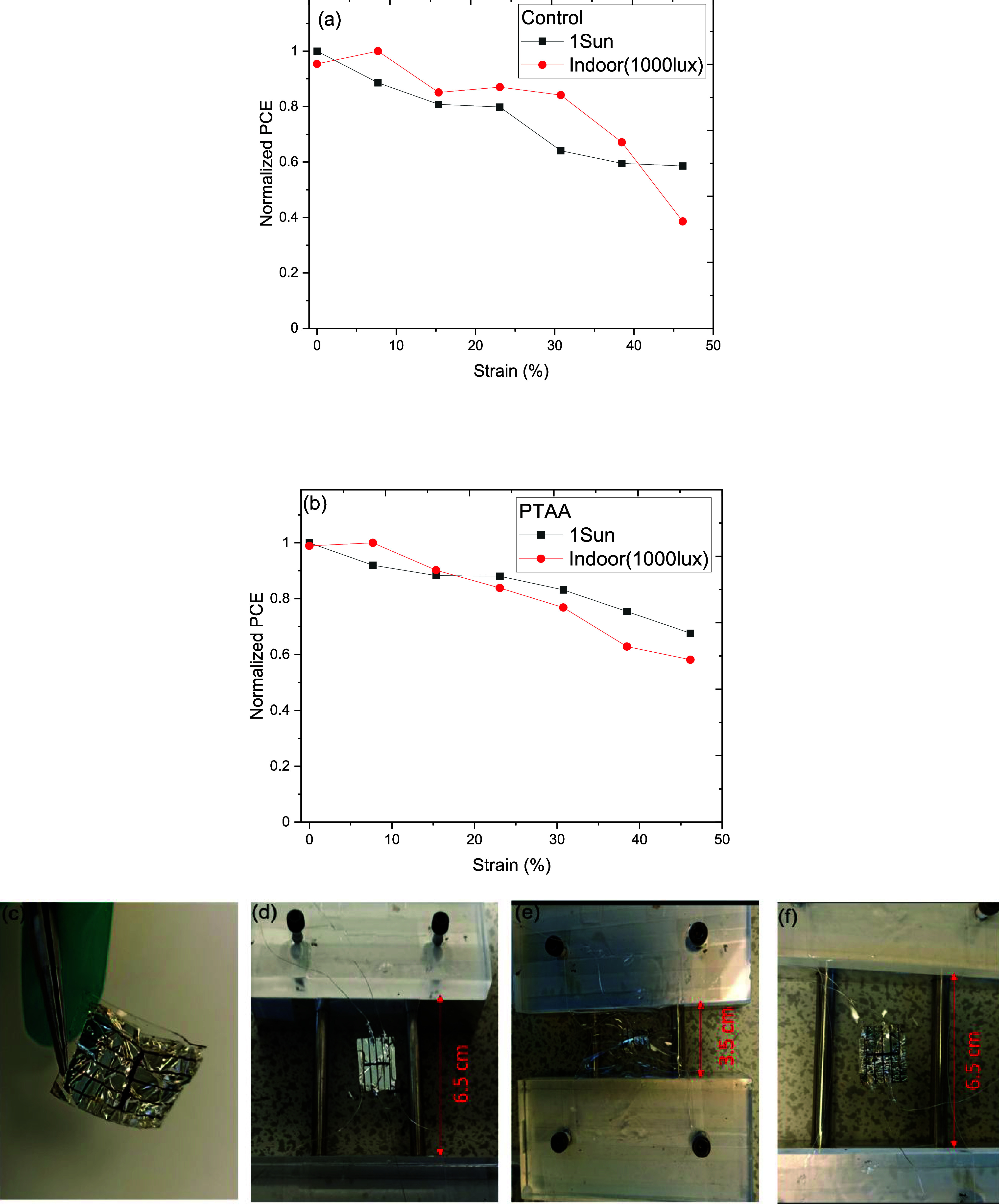
Normalized PCE of the (a) control and (b) PTAA interlayer-based devices under 1 sun and indoor LED (1000 lx) illumination. Photographs of the device substrate: (c) free-standing solar cells on PDMS/PET substrate, (d) device on prestretched elastomer, (e) device at 46.15% compressive strain, and (f) device after strain released.

Then the *J–V* characteristics of the devices were measured under indoor LED and 1 sun illumination by applying compressive strain to the devices. The devices were attached to a prestretched elastomer connected with a linear stretcher to apply compressive strain. The strain was calculated using a simplified relation: *strain* = *change in length/original length* of the elastomer. The compressive strain varied from 0 to 46.15% by decreasing the length of the prestretched elastomer by an interval of 0.5 cm. The Normalised PCE of the devices as a function of applied strain is shown in Figure 3a,b for control and PTAA interlayer-based devices, respectively. Regardless of the PTAA interlayer, the devices showed similar performance under applied strain, indicating the good mechanical compliance of the devices. The *J–V* curves of the control and PTAA interlayer-based devices measured under 1 sun and indoor LED at different applied strains are shown in Figure S4. Figure 3a–f shows the photograph of a device from free-standing to strain applied and released stage. The PSCs fabricated on the PET substrate showed impressive performance under the applied compressive and tensile strain. Under compressive strain at 30%, the device retained 83 and 76% for 1 sun and indoor light, respectively.

**Figure 4:**
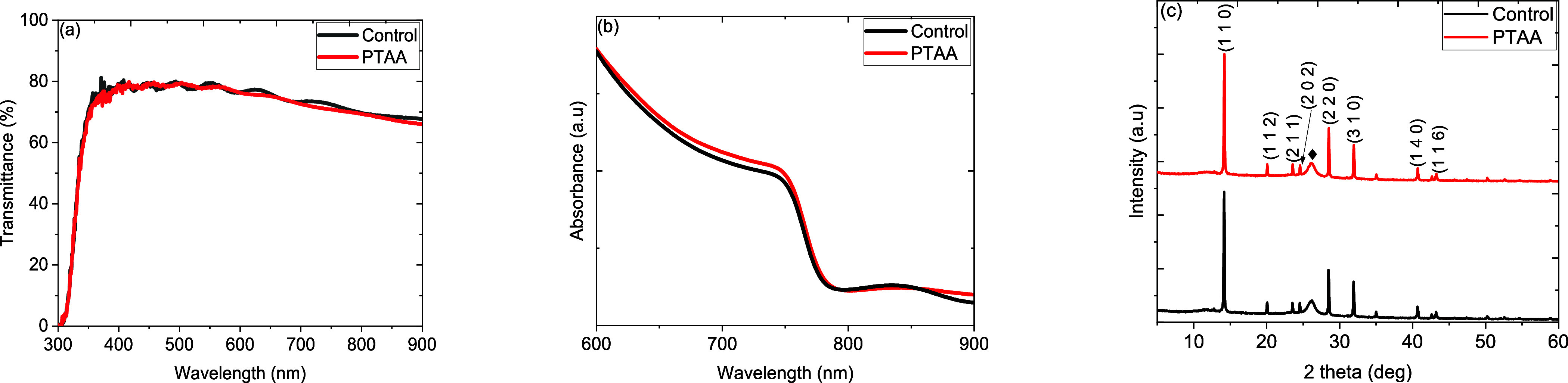
(a) Transmission and (b) absorption spectra of AI4083 and AI4083/PTAA films coated on PDMS/PET substrates. (c) XRD patterns of MAPbI_3_ perovskite layer deposited on AI4083 and AI4083/PTAA films coated on PDMS/PET substrates.

To further understand the impact of the PTAA interlayer on the enhanced performance of the devices, we studied structural, morphological, and optoelectronic characterizations of the films and devices. Figure S6 shows the scanning electron microscopy images of the MAPbI_3_ film deposited on AI4083 and AI4083/PTAA surfaces, both on the PH1000-coated PET substrate. There is no significant change observed on the surface morphology except that the film on the PTAA interlayer showed more electron beam sensitivity than on the AI4083. Further to understand whether the PTAA interlayer affects the transmittance of the light to the perovskite film, transmission spectra were recorded on PDMS/PET/PH1000/AI4083 and with PTAA. Figure 4a shows the transmittance spectra of control and PTAA films. There is no significant change in transmittance observed, indicating that the perovskite film receives the same amount of photons for charge carrier generation. Figure 4b shows the optical absorption spectra of perovskite films with and without PTAA interlayer. The absorption at 747 nm slightly increased with the PTAA interlayer; however, onset at ∼787 nm is almost the same for both the films. The XRD pattern of the perovskite films on AI4083 and PTAA surfaces is shown in Figure 4c. Both the films show preferred orientation at 14.25° corresponding to the (1 1 0) plane with secondary dominant peaks at (2 2 0) and (3 1 0) crystallographic planes. The peak at 26.21° (marked with a diamond symbol) is associated with the PET substrate. There is no change in the peak intensity observed between the films deposited on AI4083 or PTAA surfaces, indicating that the perovskite formation is not surface-dependent in this case as the concentration of the PTAA interlayer is minimal.

**Figure 5:**
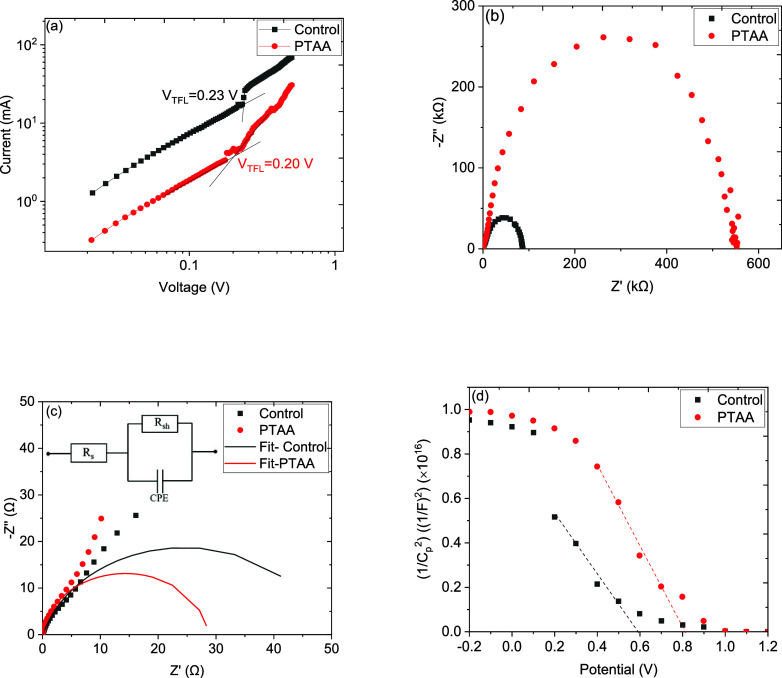
Control and PTAA interlayer-based devices for (a) dark *I*–*V* curves of HTL only devices, (b) Nyquist plot, (c) magnified view of the Nyquist plot in high-frequency region, and (d) M-S flat band potential curves.

Further to understand the impact of the PTAA interlayer and the charge transport properties of the devices, we carried out the space charge limited current (SCLC) method for hole only devices with the configuration of PET/PH1000/AI4083/MAPbI_3_/Spiro-OMeTAD/Ag and with the PTAA interlayer. The logarithmic dark *I*–*V* curves of the devices are shown in Figure 5a, where a transition from the ohmic region (linear region) at low voltage to the trap-filled (*V*_TFL_) region at higher voltage can be clearly seen according to *J* ∝ *V*^*n*^.^43,44^ The onset voltage of *V*_TFL_ and the trap density (*n*_t_) can be calculated using the Mott–Gurney relation,^45^*n*_t_ = 2*V*_TFL_εε_0_/*ed*^2^, where *d* is the thickness of the perovskite film (∼400 nm), *e* is the electron charge, ε is the dielectric constant (28.8),^46^ and ε_0_ is the vacuum permittivity. The *V*_TFL_ values fitted from the SCLC plot are 0.23 and 0.20 V for control and PTAA interlayer devices, respectively. From the Mott–Gurney equation, the *n*_*t*_ is directly proportional to V_TFL_,; therefore, the lower V_TFL_ obtained for the PTAA-based device signifies a lower concentration of the trap states. The resulting *n*_t_ calculated for control and PTAA interlayer-based devices are 4.45 × 10^16^ and 3.87 × 10^16^ cm^–3^, respectively. The reduced trap density is further evident from the improved solar cell performance under both 1 sun and indoor light.^47,48^

The electrochemical impedance spectroscopy (EIS) of the PSCs on the PET substrate was carried out to understand the charge transport properties over the frequency range of 0.1 Hz to 1 kHz with an ac amplitude of 5 mV under dark. Figure 5b shows the Nyquist plot of the control and PTTA interlayer-based devices, and a magnified view to distinguish the semicircle at the high-frequency region is shown in Figure 5c fitted with the equivalent model shown as an inset. The observed first arc is usually related to the charge transport resistance (*R*_ct_) at the interface between the perovskite and HTL, while the second arc is attributed to the charge recombination resistance within the perovskite film.^49^ From the Nyquist plot, the extracted transport resistance reveals *R*_ct_ values of 54.44 and 29.22 Ω for control and PTAA-based devices, respectively. The small *R*_ct_ is beneficial for the efficient hole extraction from the perovskite layer to the PTAA layer.^50^ EIS is further used to analyze the voltage modulation and characterize the built-in potential (*V*_bi_) using (M-S) relationship as shown in Figure 5d. The *V*_bi_ is defined by the intersection of the 1/*C*^2^ curve and the horizontal bias axis, which probes into charge accumulation at the interface between the perovskite and HTL that affects the potential barrier.^51,52^ The *V*_bi_ of the control device shows 0.59 V, while the PTAA interlayer-based device exhibits 0.80 V, respectively. The high *V*_bi_ indicates fast charge collection and reduced carrier accumulation.^53^ On the other hand, low *V*_bi_ can be attributed to the increase in the trap state densities at the interface. Based on the solar cell performance and characterization, it is evident that the PTAA interlayer enhances the hole extraction, which led to improved solar cell performance. The mechanism for the improved device performance under indoor light is that the PTAA interlayer strengthens the interfacial contact through the band alignment between the AI4083 and perovskite film and increases the shunt resistance, which suppresses the interfacial recombination and accelerates the hole transfer under indoor light operation.

## Conclusions

PSCs on deformable substrates were systematically studied for indoor light energy harvesting in addition to 1 sun illumination. We optimized the PTAA interlayer between the perovskite and HTL in order to accelerate the photogenerated hole extraction from the perovskite layer. The PTAA interlayer strengthened the interfacial contact through the band alignment and improved the shunt resistance, which suppressed the interfacial recombination and enhanced the overall device performance. Consequently, the champion device exhibited a PCE of 18.37% under 250 lx cool white LED (4000 K) light. The champion device illuminated under 250–1000 lx cool white LED panel generated a maximum power output of 13.78–25.38 μW/cm^2^. The devices were further investigated under mechanically applied compressive strain, and the PTAA-based devices showed impressive performance even after mechanical deformation and retained 83 and 76% for 1 sun and indoor light, respectively, under 30% compressive strain. This work opens up new direction to fabricate PSCs on mechanically deformable substrates for application as off-grid power source on nonplanar, curvilinear, and movable surfaces.

## Supporting Information Available

Experimental details, *J*–*V* curves and photovoltaic parameters of the different concentrations of PTAA interlayer, box chart of control and champion PTAA interlayer-based solar cell under 1 sun and 500 lx LED illumination, EQE of the control and champion PTAA interlayer-based solar cells, photovoltaic parameters of the control and PTAA interlayer-based perovskite solar cells on PET substrates measured under indoor LED at different illuminations, reverse scan *J*–*V* curves of the control and PTAA interlayer-based devices measured under 1 sun and indoor LED, and scanning electron microscopic images of the MAPbI_3_ film on control and PTAA interlayer (PDF)
