# Supplementary material for: Indoor Light Harvesting Perovskite Solar Cells on Conducting Oxide-Free Ultrathin Deformable Substrates
Source: ACS Appl Energy Mater. 2024 Jul 22;7(15):6096–104. doi: 10.1021/acsaem.3c02581 (PMC11322909; doi:10.1021/acsaem.3c02581)
Supplement: Supplementary file 1 — ae3c02581_si_001.pdf [file ae3c02581_si_001.pdf]

Supporting information

Indoor light harvesting perovskite solar cells on conducting oxide free ultrathin deformable substrates

Arivazhagan Valluvar Oli and Aruna Ivaturi*

Smart Materials Research and Device Technology Group, Department of Pure and Applied Chemistry, University of Strathclyde, Glasgow G1 1XL, United Kingdom

*Corresponding author aruna.ivaturi@strath.ac.uk

Experimental

Materials

All the materials used in this work were purchased from commercial suppliers, and used as received. These included PH1000 and AI4083 (Ossila), Poly[bis(4-phenyl)(2,5,6-trimethylphenyl)amine (M515 PTAA, 99.9%, Ossila), N,N-Dimethyl formamide (DMF, 99.8%, Acros organics), Dimethyl sulfoxide (99.7+%, Acros organics), Toluene (99.8%, Sigma-Aldrich), Chlorobenzene (99.8 % Acros organics), 2-propanal (Sigma), Lead (II) iodide (PbI_2, 99.99 %, TCI), Methylammonium iodide (MAI, 99.99%, Greatcell Solar Materials), PCBM (Luminescence Technical Corp), BCP (Ossila), Silver pellet (99.99%, Kurt. J. Lesker), PET foil (Dupont Teijin films) and Sylgard 184 Silicone Elastomer (Dow).

Characterizations

Perovskite film surface were imaged using the field-emission scanning electron microscope (FE-SEM, FEI Quanta 250 FEG). The absorption and transmittance of the films was measured using UV-Vis spectroscopy (UNICAM UV-300). The XRD measurements were performed at room temperature on a Bruker D2 Phaser system using monochromatic CuKα radiation with a wavelength of 1.5406 Å. The samples were scanned in the range 5-80° and an increment of 0.04° on the 2θ scale. The substrates were set to a rotation speed of 8 rpm throughout the measurement. Photoluminescence (PL) measurements were done using Horiba Fluorolog 3-22 with xenon lamp at an excitation wavelength of 520 nm. The Electrochemical impedance spectroscopic measurements were performed using AUTOLAB PGSTAT 302N.

The current-voltage measurements on the rigid substrate stage were carried out using Ossila I-V kit. For 1 sun measurement, the solar spectrum at AM 1.5 was simulated with a Xenon lamp and filters (Oriel Sol 1A, class ABB, 94021A) with the measured intensity at 100 mW cm^{-2}. The illumination intensity was calibrated using reference Si solar cell (Newport, 91150V). For indoor LED measurement, 4000K cool white LED panel were used. ILT350 spectrophotometer was used to measure the lux, incident power, and related spectral distribution of the LED panel. For all the light sources, current density versus voltage (J-V) curves of the resulting devices were acquired in both forward-scan (FS: -0.2 to1.2 V) and reverse scan (RS: 1.2 to -0.2V) with the scan speed of ~80 mV/s. The active area of the devices is determined by the standard aperture mask provided with the Ossila I-V kit. For the J-V measurement under compressive strain, the electrodes from the devices were connected through a copper wire and glued using silver paste and cured at 60 °C for 15 minutes. The Keithley source meter was used to measure the current-voltage output. The active area of the devices was 0.1 cm^2 and no aperture mask used.

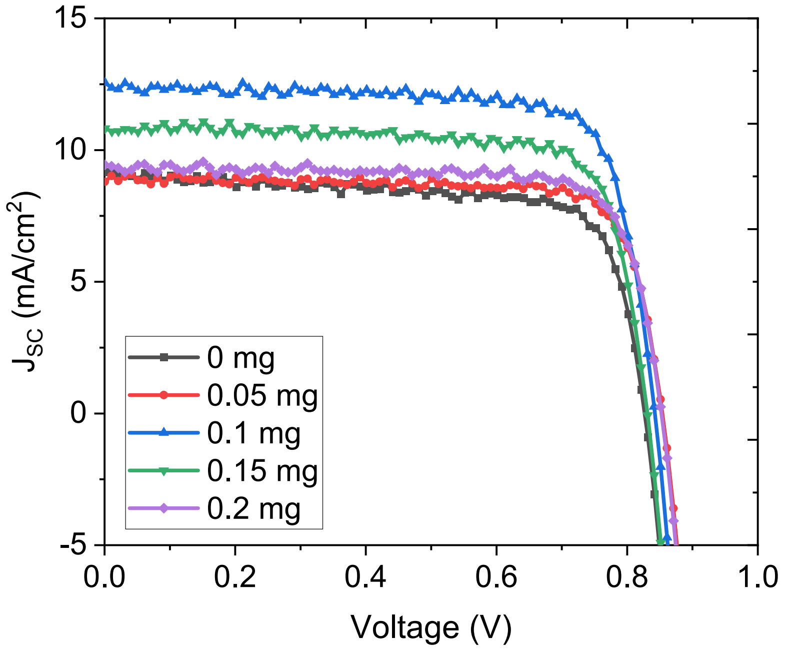

Fig. S1: Reverse scan J-V curves of the perovskite solar cells with different concentration of PTAA interlayer

Table S1: *Photovoltaic parameters of the perovskite solar cells with different concentration of PTAA interlayer*

PTAA concentration	Scan	PCE (%)	FF (%)	J_{SC}(mA/cm^2)	V_{OC} (V)
0 mg	R	5.62	74.26	9.15	0.83
	F	5.33	70.66	9.10	0.83
0.05 mg	R	6.14	80.24	8.96	0.85
	F	5.95	79.94	8.71	0.86
0.1 mg	R	8.22	78.41	12.44	0.84
	F	8.04	76.54	12.45	0.84
0.15 mg	R	7.13	78.99	10.84	0.83
	F	7.09	78.90	10.81	0.83
0.2 mg	R	6.27	78.65	9.35	0.85
	F	6.39	80.32	9.31	0.85

It is note that, the batch of devices used to optimize the PTAA concentration was different and was only considered for optimal concentration within this batch. The optimal 0.1 mg showed improved performance in another batch reported for detailed investigation in this work.

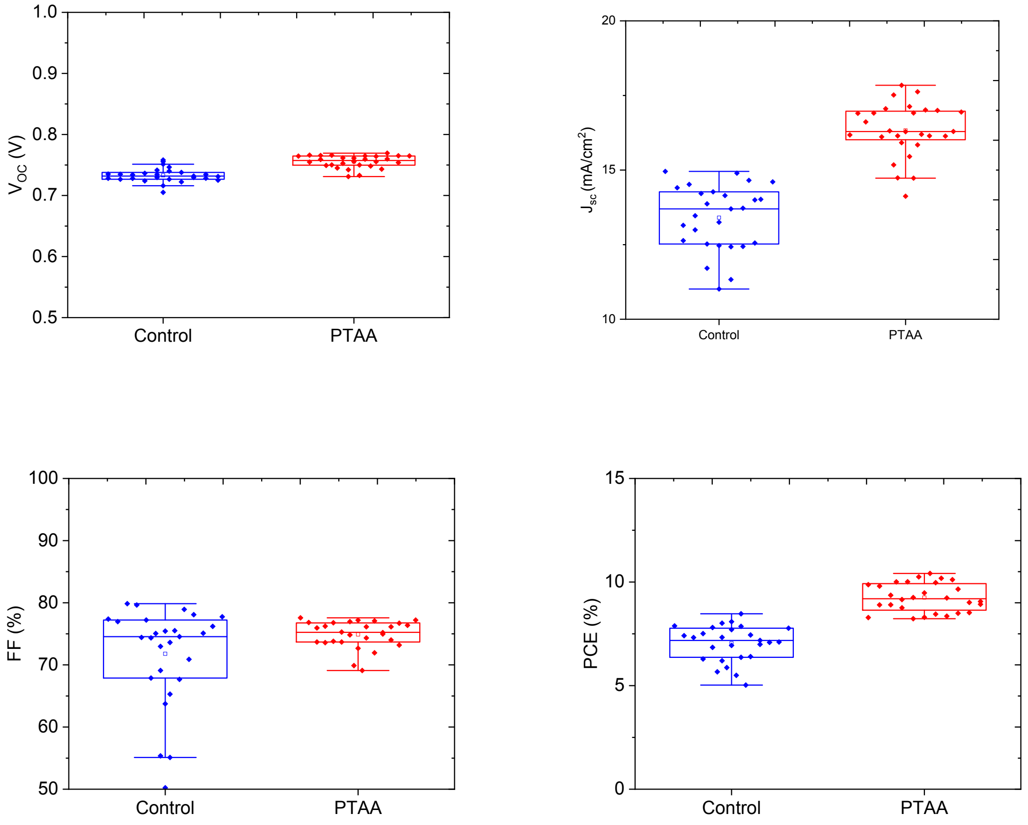

Fig. S2: Box chart of the photovoltaic parameters for the control and PTAA interlayer based devices under 1 sun illumination.

Table S2: *Photovoltaic parameters of the control and PTAA interlayer based perovskite solar cells on PET substrates measured under indoor LED panel (4000K) at different illuminations.*

Lux/intensity (~mW/cm^2)	Scan	PCE (%)	Jsc (µA/cm^2)	FF (%)	Voc (V)	P_{max} (µW/cm^2)	R_{sh} (kΩ)	R_s (Ω)
**Control**								
1000/0.300	R	5.18	47.26	54.7	0.60	15.56	33.68	47.26
	F	5.23	46.5	54.27	0.62	15.7	33.21	46.56
750/0.225	R	4.40	37.35	47.86	0.55	9.92	26.48	37.35
	F	4.64	36.41	48.12	0.59	10.46	20.90	36.41
500/0.150	R	5.57	39.67	42.12	0.50	8.37	21.16	39.67
	F	5.32	33.61	42.11	0.56	7.99	21.24	33.61
250/0.075	R	8.97	40.73	34.99	0.47	6.73	23.65	40.73
	F	8.43	34.09	34.81	0.53	6.33	26.15	34.09
**PTAA**								
1000/0.300	R	8.46	63.75	57.82	0.68	25.38	48.53	63.75
	F	8.34	63.12	57.55	0.68	25.02	49.28	63.12
750/0.225	R	9.64	61.91	51.28	0.68	21.71	57.35	61.91
	F	9.50	61.09	51.18	0.68	21.38	51.66	61.09
500/0.150	R	11.91	59.44	44.31	0.67	17.87	50.69	59.44
	F	11.55	57.89	44.12	0.67	17.33	56.524	57.89
250/0.075	R	18.37	55.62	36.99	0.66	13.78	62.159	55.62
	F	18.11	54.98	36.91	0.66	13.59	42.91	54.98

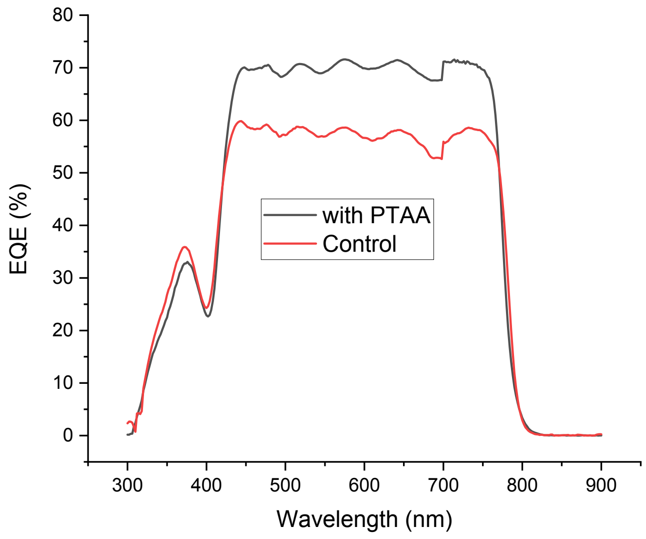

Fig. S3: EQE of the control device (red curve) and that with PTAA interlayer (black curve).

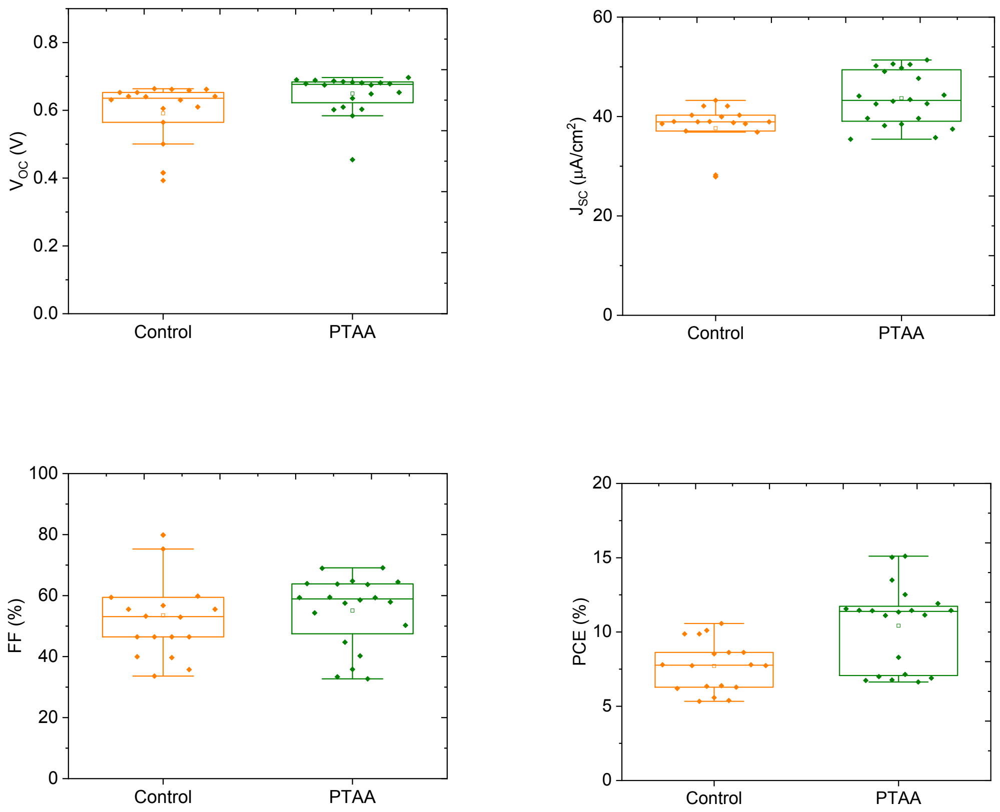

Fig. S4: Box chart of the photovoltaic parameters of the control and PTAA interlayer based devices measured under 500 lux LED illumination.

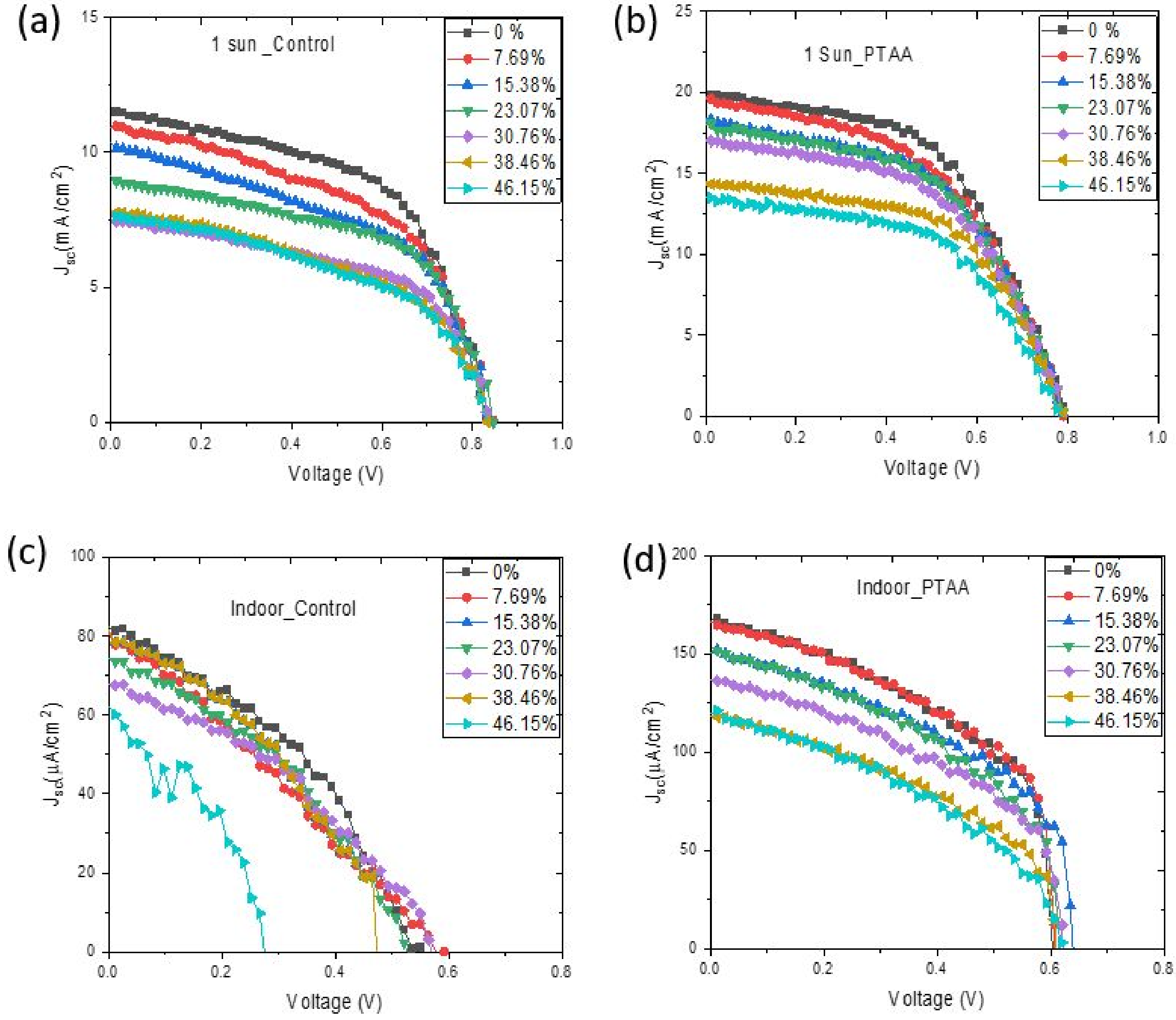

Fig. S5: Reverse scan J-V curves of the control (a, b) and PTAA interlayer (c, d) based devices measured under 1 sun and indoor LED (1000 lux) at different applied strain (0-46.15%), respectively.

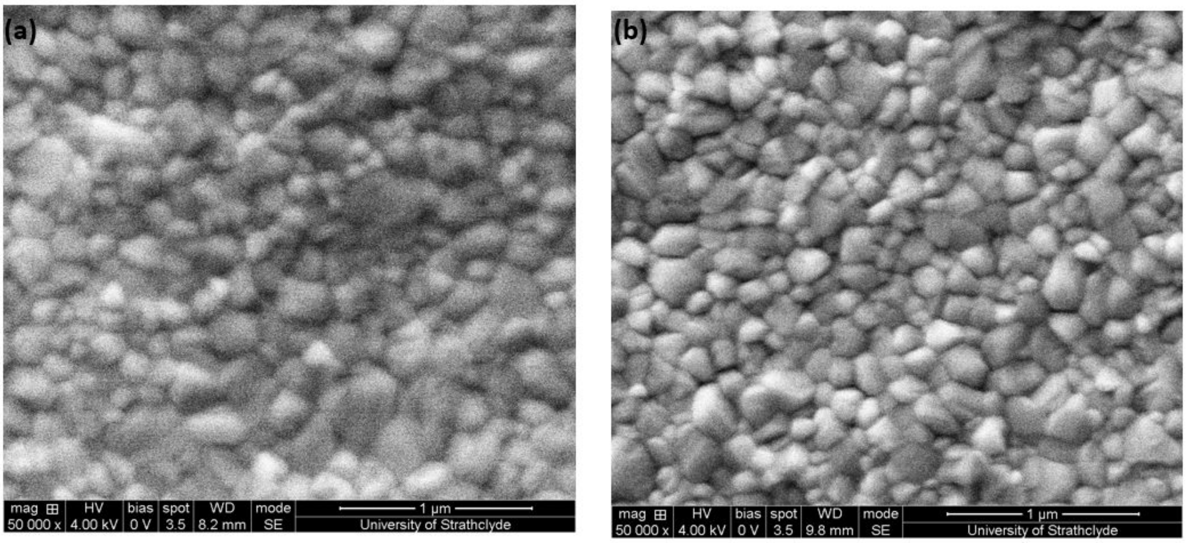

Fig. S6: Scanning electron microscopic images of the $MAPbI_3$ film on a) control and b) PTAA interlayer.
